# Effects of Early and Delayed Antiretroviral Therapy on Plasma Anti-CD4 Autoreactive IgG and Its Association With CD4^+^ T-Cell Recovery in Acute HIV-Infected Individuals

**DOI:** 10.3389/fphar.2020.00449

**Published:** 2020-04-08

**Authors:** Aixin Song, Zhen Li, Zhenwu Luo, Xiaofan Lu, Rui Wang, Lifeng Liu, Wei Xia, Zhuang Wan, Tong Zhang, Bin Su, Wei Jiang, Hao Wu

**Affiliations:** ^1^ Center for Infectious Diseases, Beijing Youan Hospital, Capital Medical University, Beijing, China; ^2^ Beijing Key Laboratory for HIV/AIDS Research, Beijing, China; ^3^ Department of Microbiology and Immunology, Medical University of South Carolina, Charleston, SC, United States; ^4^ Division of Infectious Diseases, Medical University of South Carolina, Charleston, SC, United States

**Keywords:** CD4^+^ T cells, acute HIV infection, antiretroviral therapy, autoantibody, men who had sex with men (MSM)

## Abstract

**Background:**

Plasma levels of anti-CD4 autoantibodies are increased in chronically HIV-infected patients and inversely correlated with CD4^+^ T-cell recovery under viral-suppressive antiretroviral therapy (ART). However, it remains unknown the effect of early ART on plasma anti-CD4 autoantibody levels in acute HIV infection (AHI).

**Methods:**

In this cohort study, we evaluated the effect of early and delayed initiation of ART on plasma anti-CD4 autoantibody levels in AHI individuals (n = 90). Blood samples were collected from men who had sex with men (MSM) with acute infection, pre-ART, and 4, 24, 48, and 96 weeks after ART. Plasma levels of anti-CD4 immunoglobulin G (IgG) were measured by ELISA.

**Results:**

We found that plasma anti-CD4 IgG levels were significantly increased in AHI individuals compared with healthy controls (HCs) prior to ART. Notably, early ART decreased plasma anti-CD4 IgG to the levels similar to HCs starting at 24 weeks (W). However, delayed initiation of ART did not significantly reduce plasma anti-CD4 IgG levels in AHI individuals. Moreover, the peripheral CD4^+^ T-cell counts were inversely correlated with plasma anti-CD4 IgG levels in AHI individuals at 48 and 96 W after early ART but not after delayed ART.

**Conclusions:**

Taken together, our findings demonstrate for the first time that early ART, but not delayed initiation of ART, is effective in influencing anti-CD4 autoantibody production and recovering CD4^+^ T-cell counts in AHI individuals.

## Introduction

Human immunodeficiency virus (HIV) infection results in progressive depletion of CD4^+^ T cells, which leads to immune perturbations and severe opportunistic infections. Antiretroviral therapy (ART) suppresses viral replication, increases CD4^+^ T-cell counts and slows HIV disease progression (Battegay et al., 2006; May et al., 2006; Emery et al., 2008). However, a small proportion of patients fails to reconstitute their peripheral CD4^+^ T-cell counts even with long-term viral-suppressive ART (Nakanjako et al., 2008; Kelley et al., 2009). Notably, the mortality and incidence of osteoporosis, cardiovascular, liver, and kidney diseases are increased in patients with blunted CD4^+^ T-cell recovery under ART (Deeks et al., 2013; Lapadula et al., 2013).

HIV infection is associated with humoral immune perturbations such as B-cell hyperactivation and hypergammaglobulinemia prior to ART (Zandman-Goddard and Shoenfeld, 2002; Iordache et al., 2014; Moody et al., 2016). ART partially reduces B-cell polyclonal activation and autoantibody production; however, hypergammaglobulinemia is not fully controlled in some patients even under viral-suppressive ART (Zandman-Goddard and Shoenfeld, 2002; Satta et al., 2018). The levels of anti-apoA-1 immunoglobulin G (IgG) are increased in chronic HIV-infected individuals and are associated with lower CD4^+^ T-cell counts and levels of systemic inflammation; *in vitro* treatment with anti-apoA-1 IgG induced dose and time-dependent CD4^+^ T-cell apoptosis (Satta et al., 2018). The antigen-specific IgG produced by B-cell plays a vital protective role in the immune response against pathogens in HIV infection, and IgG accounts for about 70–75% of the total immunoglobulin. In the absence of ART, B cell polyclonal activation and increased autoantibody production have been observed in HIV-infected individuals at both the acute phase (AHI) and chronic phases. After ART, most polyclonal B-cell activation and elevated autoantibody levels can reduce to the levels similar to healthy controls (HCs). Recently, we reported that the levels of anti-CD4 IgG are elevated in immune non-responders (aviremic, ART-treated, and CD4^+^ T-cell counts < 350 cells/µl) and anti-CD4 IgG purified in plasma from non-responders activates NK cells and induces CD4^+^ T-cell apoptosis through antibody-dependent cellular cytotoxicity (ADCC) (Lederman et al., 2011). Moreover, the percentage of surface auto-IgG on CD4^+^ T cells is associated with the percentage of CD4^+^ T-cell apoptosis and CD4^+^ T-cell counts under viral-suppressive ART (Luo et al., 2017a; Luo et al., 2017b). In an animal model, plasma levels of autoreactive antibodies against CD4^+^ T cells, but not anti-CD4 autoantibodies, was associated with progressive decline of CD4^+^ T cells in simian immunodeficiency virus (SIV)-infected rhesus macaques; and this association was observed in non-SIV animal models with immune activation and autoimmunity (Kuwata et al., 2009). Therefore, elevated plasma anti-CD4 IgG levels may reveal an important mechanism of insufficient immune reconstitution in chronically HIV-infected individuals with viral suppression under ART. Intriguingly, elevated anti-CD4 antibodies were found in plasma from HIV patients after seroconversion or prior to seroconversion, and even in plasma from HIV seronegative patients (Callahan et al., 1992; Keiser et al., 1992). Nowadays, early ART was recommended to initiate in primary HIV-infected patients, and studies revealed that patients initiated ART within 3–6 months after HIV infection enhanced CD4^+^ T-cell recovery and reduced chronic immune activation (Kaufmann et al., 2005; Le et al., 2013; Sun et al., 2017). However, the effects of early ART on plasma levels of anti-CD4 IgG in AHI individuals have not been reported.

Several mechanisms, such as persistent inflammation, fibrosis of thymus and lymphoid tissues, and gut mucosal dysfunction, are considered as factors for poor CD4^+^ T-cell recovery after viral-suppressive ART (Diaz et al., 2010; Kingkeow et al., 2015). Importantly, studies from others and from our team reveal that anti-CD4 autoantibodies play a role in CD4^+^ T cells depletion in ART-treated chronic HIV and SIV infection (Dalgleish, 1995; Kuwata et al., 2009; Luo et al., 2017a; Luo et al., 2017b). Moreover, elder age, longer duration of HIV infection and lower pre-ART CD4^+^ T-cell counts are associated with incomplete recovery of CD4^+^ T cells (Kaufmann et al., 2005; Stirrup et al., 2018). In addition, low nadir CD4^+^ T-cell counts and elevated CD4^+^ T-cell activation are associated with poor CD4^+^ T-cell recovery (Hunt et al., 2003; Lederman et al., 2011). However, the CD4^+^ T-cell recovery and factors associated with CD4^+^ T-cell recovery after early ART remain unclear.

In the current study, we aim to investigate the dynamic production of plasma levels of anti-CD4 IgG in AHI individuals following early and delayed initiation of ART. In addition, total IgG and antinuclear antibody (ANA) have been evaluated as well. We found that plasma levels of anti-CD4 IgG are significantly elevated in AHI individuals, and early ART rather than delayed ART normalizes plasma anti-CD4 IgG levels starting at 24 W after treatment.

## Materials and Methods

### Study Subjects

This was a retrospective study. Ninety acute HIV-infected individuals (AHI) were enrolled from the Beijing PRIMO clinical cohort established by Beijing Youan hospital, Beijing, China (Huang et al., 2013; Li et al., 2017). In this cohort, HIV-negative men who had sex with men (MSM) were recruited and followed up every 2–3 months; plasma levels of HIV RNA and HIV-specific antibody were detected at each visit until seroconversion. AHI was defined as a positive result of HIV RNA but a negative or indeterminate HIV-specific antibody status (Chin et al., 2013). The progression of AHI can be described as six discrete stages proposed by Fiebig *et al.* (Fiebig et al., 2003; Cohen et al., 2010). The control group was selected from the repository samples who have not been infected. In the current study, there were seven cases at Fiebig stage I and three cases at Fiebig stage II, the rest of patients were at stage V–VI. AHI individuals were divided into early ART (n = 69) and delayed ART (n = 21) group according to the time of ART initiation. Patients on early ART group (TDF+3TC+EFV or TDF+3TC+LPV/r) were immediately treated once they were identified as AHI, and the estimated infection time was 69.4 ± 4.6 (mean ± standard error of mean, SEM) days. Patients on delayed ART group (TDF+3TC+EFV or AZT+3TC+NVP) were treated at the chronic infection stage, and the estimated infection time was 456.7 ± 48.4 days. All the individuals were followed up at 0, 4, 24, 48 and 96 W after ART. CD4^+^ T-cell count and plasma HIV RNA level were measured at each visit. Inclusion criteria of AHI subjects were age above 18 years and not having ART prior to enrollment. Exclusion criteria were opportunistic infections and co-infections with hepatitis B virus, hepatitis C virus, or tuberculosis. Forty age-matched HIV-negative MSM individuals were served as HCs. The clinical characteristics of all subjects were shown in **Table 1**.

**Table 1:** Characteristics of all the study participants.

Characteristics	Healthy controls	Early ART group	Delayed ART group	*P* value
Cases	40	69	21	
Sex, male (%)	40 (100%)	69 (100%)	21 (100%)	0.99
Age (y)	29 (20-46)	31 (18-54)	31 (22-45)	0.25
Infection time before ART (day)	NA	69.4 ± 4.6	456.7 ± 48.4	<0.0001
CD4^+^ T-cell count, cells/µL	916 (318-1466)	409 (174-1033)	242 (91-328)	<0.0001
Nadir CD4^+^ T-cell count, cells/µL	NA	347 (135-717)	234 (91-328)	<0.0001
Plasma viral load (log10 copies/mL)	NA	4.5 (2.6-7)	4.2 (1.7-5.9)	0.37
Drugs	NA			
TDF+3TC+EFV		65 (94%)	10 (48%)	
TDF+3TC+LPV/r		4 (6%)		
AZT+3TC+NVP			11 (52%)	

Data were presented as median (interquartile range).

P values were determined by Mann–Whitney test.

NA, not applicable.

This study has been approved by the Beijing Youan Hospital Research Ethics Committee and all participants provided written informed consents. The methods were carried out in accordance with the approved guidelines.

### Enzyme-Linked Immunosorbent Assay (ELISA) for Detection of Anti-CD4 IgG

Human soluble CD4 protein (sCD4; Progenics, Tarrytown, NY) was diluted (16 μg/ml) and added into 96-wells microtiter plates and incubated at 4°C overnights. Microwells were then washed three times with phosphate-buffered saline (PBS) wash buffer (PBS with 0.1% Tween 20). The plate was blocked by PBS containing 3% bovine serum albumin (BSA) and incubated for 120 min at 37°C. Plasma was diluted in 1:40 in PBS and 100 μl of the dilution was added to each well. The plate was incubated at room temperature (RT) with rotation at 600 rpm/min for 60 min. Goat anti-human IgG-HRP was diluted in 1:5,000 in PBS and added to the microtiter wells with an incubation period of 60 min at RT. After washing three times, 100 μl of 2′-Azino-di (3-ethylbenzthiazoline-6-sulfonate) (ABTS, KPL, Cat#50-66-00) was added to each well. Then incubated in dark for 30 min at RT, stop solution was added into the microwells to stop the enzyme reaction. Optical density was determined at 405 nm within 30 min using a microplate reader (Luo et al., 2017b).

### ELISA for Detection of Total IgG

Plasma levels of human IgG was quantified using a commercial kit (Beijing Jinhao Medical Company, Beijing, China). All kits reagents and materials reached to RT (20–25°C) for 30 min before use. Added 50 μl of standards and samples in appropriate microtiter wells, then 100 μl of Enzyme conjugate was added to standard and sample wells, covered with an adhesive strip and incubated for 60 min at 37°C. Microwells were then washed five times with wash solution (dilute 1 volume of wash solution 20X with 19 volume of distilled water). Added substrate A 50 μl and substrate B 500 μl to each well. Gently mixed and incubated protect from light for 15 min at 37°C. Stop solution was added into the microwells and the color in the wells changed to yellow. Optical density was determined at 450 nm using a microplate reader within 15 min.

### Indirect Immunofluorescence Testing (IIFT) for Detection of ANA IgG

Plasma levels of ANA IgG detection was performed using a commercial kit according to the manufacturer’s protocol by indirect immunofluorescence assay on tissue mosaic slides (Euroimmun, Lübeck, Germany). Briefly, Serum samples were diluted from 1:100, Hep-2-coated slides were incubated for 30 min with 32 μl diluted plasma, after washing incubated with 20 μl FITC-labelled goat anti-human IgG for 30 min. All incubations were performed at room temperature (18–25°C). ANA-positive and ANA-negative controls were included in experiments. The enzymatic reaction was stopped by washing with PBS, samples were examined with a confocal microscope. The staining of patient samples was determined by comparison with the controls at equal exposure times.

### Serological Assays

HIV-1 infection status was determined by screening with a HIV-1/2 antigen/antibody combo enzyme immunoassay (Beijing Wantai Biological Medical Company, Beijing, China). Positive specimens were further conformed by using Western blot for HIV-1/2 antibodies (HIV Blot 2.0 MP Diagnostics, Singapore) (Li et al., 2017).

### Plasma HIV-1 RNA Levels and CD4^+^ T-Cell Counts

CD4^+^ T-cell absolute count (cells/μl) were determined in fresh whole blood samples from AHI individuals using Trucount Tubes and CD45^+^/CD3^+^/CD4^+^/CD8^+^ four-color antibody (BD Biosciences) by flow cytometry. Plasma HIV viral load (copies/ml) was quantified by automated Real-Time PCR M2000 system (Abbott Molecular Inc., Des Plaines, IL, USA), with a detection limit of 40 copies/ml.

### Statistical Analysis

Normal distribution data was presented as mean ± SEM, whereas abnormal distribution data was presented as median and interquartile range (IQR). Statistical analysis (including normality testing) and graphical presentation were performed using GraphPad Prism software version 6.02 (GraphPad Software, San Diego, CA, USA) and SPSS (Version 23; IBM, Armonk, NY, USA). Statistical significance between groups was determined by the non-parametric Mann-Whitney *U* test or one-way ANOVA test. Correlations between pairs of variables (CD4^+^ T-cell counts and anti-CD4 IgG levels) were analyzed by Spearman’s rank correlation test. All tests were two-tailed, and *P* < 0.05 was considered statistically significant.

## Results

### Clinical Information

We enrolled ninety AHI individuals in this study. The estimated infection time was 60.6 ± 4.0 days. All individuals were separated into early ART group (n = 69) and delayed ART group (n = 21). Forty HIV-negative individuals were recruited as HCs. The clinical characteristics of all subjects were shown in **Table 1**. The absolute CD4^+^ T-cell counts and nadir CD4^+^ T-cell counts were significantly lower in delayed ART group than those in early ART group (*P* < 0.001).

### Plasma Levels of Anti-CD4 IgG Were Dramatically Increased in Acute HIV-Infected Individuals

**Figure 1:**
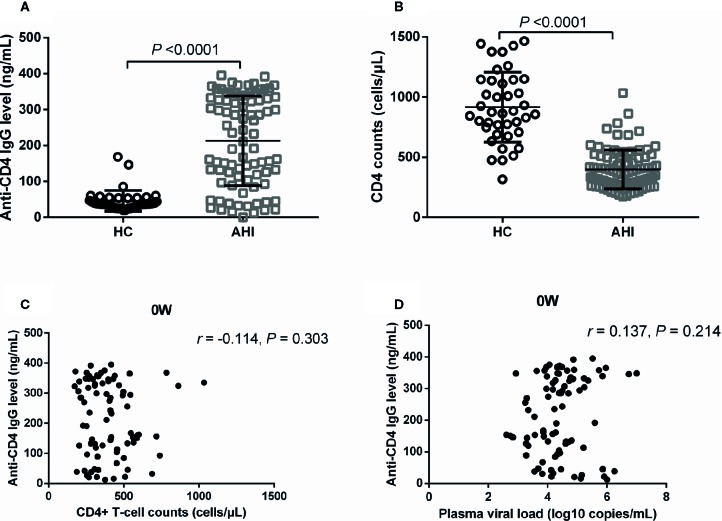
Plasma Anti-CD4 IgG Levels and CD4^+^ T-cell Counts in HCs and ART-naive AHI Individuals. Plasma anti-CD4 IgG levels **(A)** and CD4^+^ T-cell counts **(B)** in healthy controls (n = 40) and AHI individuals (n = 90) prior to ART. Correlations between plasma anti-CD4 IgG levels and peripheral CD4^+^ T-cell counts **(C)** and plasma HIV RNA levels **(D)** in AHI individuals prior to ART. Non-parametric Mann-Whitney tests and Spearman’s rank tests, with *P* < 0.05 considered significant.

Auto-reactive antibodies have been observed in both healthy people and individuals with infectious diseases (Shirai et al., 1992; Elkon and Casali, 2008; Bonsignori et al., 2014). Previous studies reported that plasma anti-CD4 IgG levels were elevated in individuals with HIV seroconversion and with chronic HIV infection (Callahan et al., 1992; Keiser et al., 1992; Luo et al., 2017b). In the current study, we found that plasma levels of anti-CD4 IgG were significantly increased in AHI individuals compared with HCs, as shown in about five-time increase (*P* < 0.0001, **Figure 1A**). CD4^+^ T-cell counts were significantly decreased in AHI individuals compared with HCs (*P* < 0.0001, **Figure 1B**). However, we did not observe any correlation between plasma levels of anti-CD4 IgG and CD4^+^ T-cell counts in ART-naive (0W) AHI individuals (**Figure 1C**). Furthermore, there was no correlation between plasma levels of anti-CD4 IgG and plasma HIV RNA levels in ART-naive AHI individuals (**Figure 1D**).

### Dynamic Changes of Anti-CD4 IgG in AHI Individuals After Early ART

**Figure 2:**
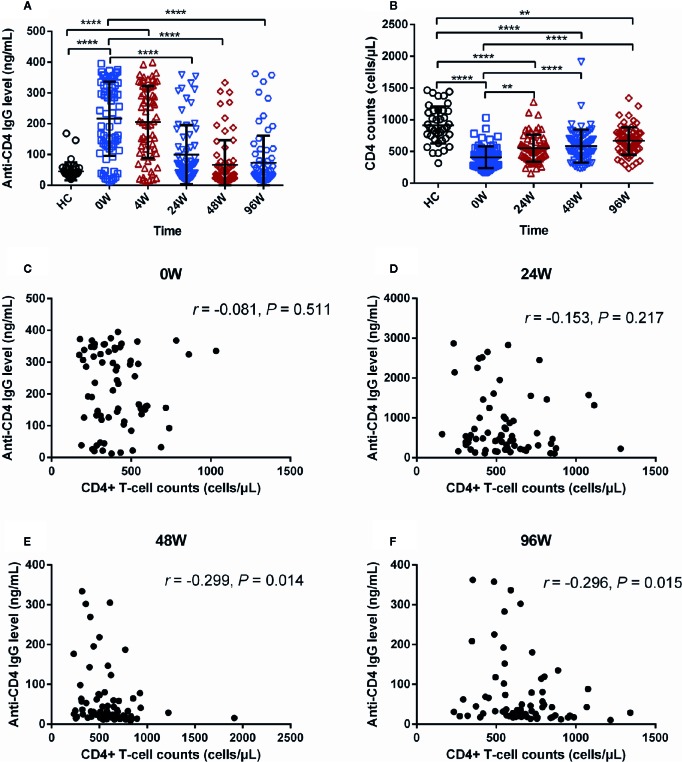
The Dynamic Changes of Anti-CD4 IgG Levels in AHI Individuals After Early ART. Plasma anti-CD4 IgG levels **(A)** at 0 (□), 4 (△), 24 (▽), 48 (◇), and 96 W (○), and CD4^+^ T-cell counts **(B)** in healthy controls (n = 40) and AHI (n = 69) individuals at 0 (□), 24 (△), 48 (▽), and 96 W (◇) after early ART. Correlations between plasma levels of anti-CD4 IgG and peripheral CD4^+^ T-cell counts in AHI individuals at 0 **(C)**, 24 **(D)**, 48 **(E)**, and 96 W **(F)** after early ART. One-way ANOVA tests and Spearman’s rank correlation tests, with *P* < 0.05 considered significant. **P < 0.01, ****P < 0.0001.

To investigate the effect of early ART on plasma levels of anti-CD4 IgG in AHI individuals, we quantified the plasma levels of anti-CD4 IgG at baseline (0), 4, 24, 48 and 96 W after early ART. As shown in **Figure 2A**, the plasma levels of anti-CD4 IgG were dramatically reduced at 24, 48, and 96 W compared with 0 W, and reached the lowest level from 48 W after early ART (*P* < 0.001). However, there was no significant difference between 0W and 4W after early ART (**Figure 2A**). The levels of anti-CD4 IgG were similar between AHI individuals at 24, 48, and 96W after early ART and HCs (**Figure 2A**). These results indicate that early ART can normalize the plasma levels of anti-CD4 IgG in AHI individuals. Meanwhile, we compared CD4^+^ T-cell counts before and after early ART and found that CD4^+^ T-cell counts were significantly increased in AHI individuals at 24, 48, and 96 W after early ART compared with those at 0 W (**Figure 2B**). However, even if CD4^+^ T-cell counts have increased at 24, 48, and 96 W after early ART; they were still significantly lower compared with those in HCs (*P* < 0.01).

Next, we have analyzed the relationships between the levels of anti-CD4 IgG and CD4^+^ T-cell counts at each time points after early ART in AHI individuals. We did not find correlations between plasma anti-CD4 IgG levels and CD4^+^ T-cell counts at 0 (**Figure 2C**) and 24 W (**Figure 2D**). Intriguingly, we found that the levels of anti-CD4 IgG were negatively correlated with CD4^+^ T-cell counts at 48 W (*r* = −0.299, *P* = 0.014, **Figure 2E**) and 96W (*r* = −0.296, *P* = 0.015, **Figure 2F**). Taken together, these findings suggest that immediate initiation of ART during AHI reduce plasma levels of anti-CD4 IgG to the levels similar in HCs, and that plasma anti-CD4 IgG levels inversely correlated with peripheral CD4^+^ T-cell counts after at least one year of early ART in AHI individuals.

### Changes of Anti-CD4 IgG in AHI Individuals With Delayed ART

**Figure 3:**
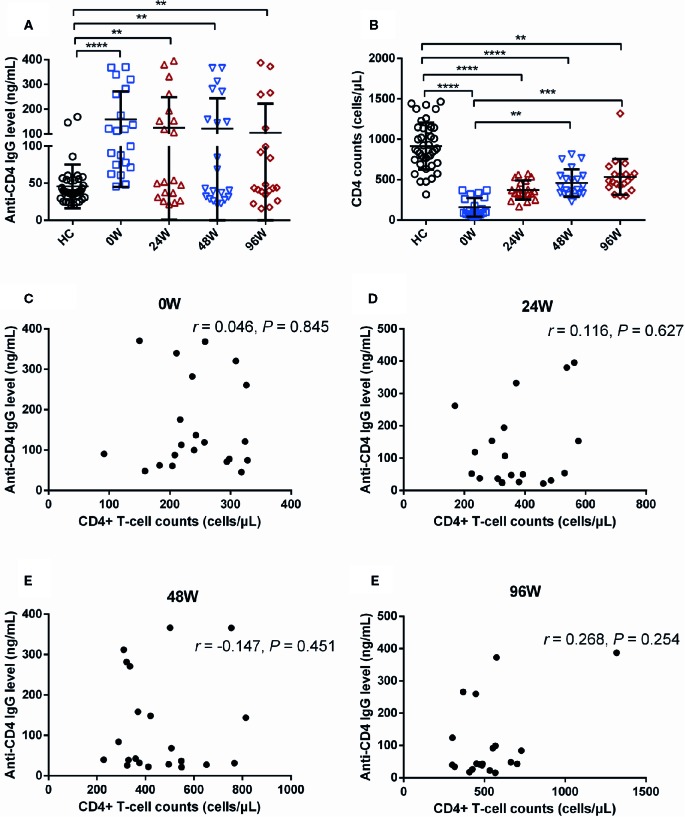
Effect of Delayed Initiation of ART on Plasma Levels of Anti-CD4 IgG in AHI Individuals. Plasma anti-CD4 IgG levels **(A)** and CD4^+^ T-cell counts **(B)** in healthy controls (n = 40) and AHI (n = 21) individuals at 0 (□), 24 (△), 48 (▽), and 96 W (◇) after delayed initiation of ART. Correlations between plasma levels of anti-CD4 IgG and peripheral CD4^+^ T-cell counts in AHI individuals at 0 **(C)**, 24 **(D)**, 48 **(E)**, and 96 W **(F)** after delayed initiation of ART. One-way ANOVA tests and Spearman’s rank correlation tests, with *P* < 0.05 considered significant. **P < 0.01, ***P < 0.001, ****P < 0.0001.

Next, we explored whether delayed initiation of ART affects plasma levels of anti-CD4 IgG. We quantified plasma levels of anti-CD4 IgG in individuals who initiated ART at chronic stage of HIV infection. Unlike early ART, levels of anti-CD4 IgG in delayed ART group were not significantly declined at each post-ART visit compared with 0 W (**Figure 3A**). Notably, levels of anti-CD4 IgG were significantly higher in delayed ART group at 24, 48, and 96 W compared with HCs (*P* < 0.01, **Figure 3A**). Furthermore, CD4^+^ T-cell counts were significantly increased in delayed ART group at 48 W, and 96 W compared with those at 0 W (**Figure 3B**). However, CD4^+^ T-cell counts in individuals who received delayed ART remained significant lower compared with HCs (*P* < 0.01, **Figure 3B**). We did not observe any correlations between anti-CD4 IgG levels and CD4^+^ T-cell counts in AHI individuals with delayed ART at 0, 24, 48, and 96 W (**Figures 3C–F**). Our findings indicate that unlike early ART, delayed ART failed to reduce anti-CD4 IgG levels to the levels similar to HCs.

### Dynamic Changes of Total IgG and ANA in AHI Individuals After Early and Delayed ART

**Figure 4:**
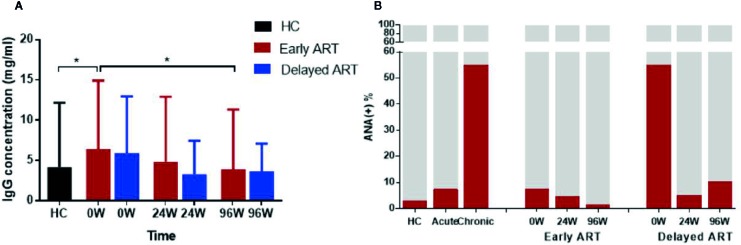
Dynamic Changes of Total IgG and ANA in AHI Individuals After Early and Delayed ART. Plasma total IgG levels **(A)** and ANA positive rate **(B)** in healthy controls (n = 40) and AHI individuals (n = 90) after early and delayed ART. One-way ANOVA tests with *P* < 0.05 considered significant. *P < 0.05.

IgG antibody plays an important role in immune response of HIV infection. The production of HIV membrane protein and capsid protein-specific IgG in different time of the early stage of infection is due to individual differences. However, the plasma levels of total IgG in AHI as well as the influence of early and delayed ART on them remain to be analyzed. In our study, the results showed that the plasma levels of total IgG were significantly increased in ART-naïve AHI individuals compared with HCs (*P <* 0.05, **Figure 4A**). Total IgG levels decreased to the levels similar in HCs after 96 W early ART with statistically significant (*P*=0.028). There was no significant difference before and after ART in delayed ART group.

Besides anti-CD4 autoantibody in HIV-infected patients, there are other autoreactive antibodies in these individuals. Indeed, plasma levels of ANA after early and delayed ART were detected in this study for comparison. We found that the ANA positive rate was significantly higher in HIV-infected patients than HC group. ANA positive rate was about 3, 8, and 50% in the control group, acute stage and chronic infection respectively (**Figure 4B**). Both early and delayed ART effectively reduce the plasma levels of ANA in AHI, the ANA positive rate reached the normal levels (2%) after 96 W early ART, while in delayed ART group, this rate (10%) was still higher comparing to HCs (**Figure 4B**). Consistent with anti-CD4 IgG, early treatment can normalize plasma ANA levels in AHI while delayed treatment cannot.

## Discussion

Autoantibodies and autoimmunity present in HIV disease (Iordache et al., 2014). Previous studies have shown that plasma autoantibody levels were inversely correlated with CD4^+^ T-cell counts or disease progression, especially with increased serum levels of tumor necrosis factor-α (TNF-α) and interferon-α (IFN-α) (Muller et al., 1993). We have reported that levels of anti-CD4 IgG in immunologic non-responders were elevated and associated with blunted CD4^+^ T-cell recovery in chronic HIV-infected patients with viral suppression after ART (Luo et al., 2017b). However, plasma levels of anti-CD4 IgG in acute HIV-infected individuals remain unclear. In this study, we reported that plasma level of anti-CD4 IgG was significantly increased in AHI individuals compared with HCs (**Figure 1**). The increase of anti-CD4 IgG in acute HIV infection may attribute to the increase of autoantigens, such as debris arise from massive CD4^+^ T-cell apoptosis, sCD4, or HIV protein binding CD4 complex (Lederman et al., 2011; Koppensteiner et al., 2012; Luo et al., 2017b). Moreover, AHI induced epithelial apoptosis and mucosal barrier dysfunction, which results in microbial translocation, immune activation, inflammation, and autoantibodies production (Shirai et al., 1992; Petta et al., 2018; Xu et al., 2018).

Early ART is recommended to prevent HIV transmission and to prevent CD4^+^ T-cell decline (Le et al., 2013; Group et al., 2015). Herein, we compared anti-CD4 IgG levels and CD4^+^ T-cell counts in AHI individuals after early or delayed ART. We found that anti-CD4 IgG levels were increased in both acute and chronic HIV infection, and that early ART significantly decreased levels of anti-CD4 IgG in AHI individuals, similar to the levels of anti-CD4 IgG in HCs since 24 W post-ART (**Figure 2**). However, there were no significant differences of anti-CD4 IgG levels in AHI individuals with delayed ART compared with those at pre-ART. These findings demonstrate that early ART rather than delayed ART can normalize anti-CD4 IgG levels in AHI individuals. Acute infection was associated with significantly lower viral and less immune activation as well as a better CD4^+^ T-cell counts compared to chronic stages, suggesting that initiating early ART may be more beneficial in mitigating HIV persistence, attenuates inflammation, rapid recovery of CD4^+^ T-cell counts, and protecting immune function. Early ART significantly enhanced probability of immunological recovery, which has not been observed in delayed ART (Ananworanich et al., 2016; Sereti et al., 2017). In addition, we found inversely correlations between plasma anti-CD4 IgG levels and CD4^+^ T-cell counts at 48 and 96 W in early ART group but not in delayed ART group. This is not consistent with our previous study, which showed that anti-CD4 IgG levels was inversely correlated to CD4^+^ T-cell counts in chronic HIV-infected individuals under ART (Luo et al., 2017b). The differences between the results in the current study and previous study may be due to the different subjects, nationality and duration of infection and ART. In this study, our subjects were AHI individuals, Chinese, and all of them were male. Subjects in previous study were Caucasians and Afro-Americans, both males and females, and with many years of infection. The duration of ART was two years in the current study and more than 5–10 years from the previous study (Luo et al., 2017b).

AHI induced sharply depleted of CD4^+^ T-cell counts (Krebs and Ananworanich, 2016). Our findings showed that either early ART or delayed ART can only partially restore the CD4^+^ T-cell counts (**Figures 2B** and **3B**). Several mechanisms may cause the blunted recovery of CD4^+^ T cells, including persistent inflammation, gut mucosal dysfunction, fibrosis of thymus, and lymphoid tissue (Hunt et al., 2003; Marchetti et al., 2008; Diaz et al., 2010; Lederman et al., 2011; Mavigner et al., 2012). In addition, increased levels of anti-CD4 IgG from non-responders induced CD4^+^ T cells apoptosis and play a role in poor CD4^+^ T-cell recovery in chronic HIV-infected individuals under ART (Luo et al., 2017a). The previous study suggests that increase of anti-CD4 IgG levels may be a novel mechanism for CD4^+^ T cell depletion in ART-treated chronically infected individuals. In the current study, we did not observe correlations between anti-CD4 IgG levels and peripheral CD4^+^ T-cell counts in ART-naive AHI individuals. Intriguingly, we found inverse correlations between CD4^+^ T-cell counts and anti-CD4 IgG in early ART-treated individuals starting at 24 W of treatment (**Figures 2E, F**). These results may imply that the anti-CD4 IgG may not be functional in untreated AHI individuals and may play a pathogenic role during immune recovery following ART but needs further investigations. Moreover, persistent immune activation and residual inflammation after ART may play a role in B cell polyclonal activation and autoantibody production during immune reconstitution. Indeed, autoimmune diseases mainly occur in the immunologic reconstitution phase after ART in chronically infected patients (Zandman-Goddard and Shoenfeld, 2002; Iordache et al., 2014). The mechanisms of pathogenic anti-CD4 IgG production remain unknown, and persistent immune activation and inflammation after ART may contribute to the breakdown of tolerance (Xu et al., 2018). Furthermore, autoantigens from apoptotic CD4^+^ T cells, sCD4, or released HIV protein-bound CD4, may provide the antigen stimulation for generation of pathologic autoantibodies in post-ART HIV-infected individuals (Luo et al., 2017a). However, the exact mechanisms need further investigations. Moreover, poor recovery of CD4^+^ T-cell counts after early ART was associated with higher anti-CD4 IgG levels. Therefore, inhibitors that target anti-CD4 IgG binding sites may at least partially prevent CD4^+^ T-cell depletion. The development of anti-CD4 IgG inhibitors together with ART may significantly improve CD4^+^ T-cell recovery.

Furthermore, a series of immunological methods and techniques based on serological changes of IgG play a vital role in HIV antibody detection, and the proportion of HIV-specific IgG and total IgG can be used to distinguish new and previous infection and calculate the incidence (McDougal et al., 2006; Jiang et al., 2007). Our findings suggested that IgG concentration was elevated in HIV-infected individuals compared to healthy people. Plasma total IgG levels could be restored to normal levels after early ART, which was consistent with the change of anti-CD4 IgG. Analysis of the dynamic changes can be used as a biomarker for HIV infection progression and contribute to vaccine development. In this study, ANA with a relatively high positive rate was selected for detection and observation and used as a reference indicator for autoantibody changes. The ANA positive rate of HIV-infected patients was higher than HCs. Both early and delayed ART can effectively reduce plasma ANA levels in HIV-infected individual. However, there are some limitations in our study. First, females tend to have increased autoantibodies and risk of autoimmune diseases compared to males, however, there were no female participants in this MSM cohort (Gilman et al., 2018). Second, the relatively small sample size and short duration of ART may limit the power to obtain the desired effect. Third, the different ART regimen may affect the results. Finally, the delayed ART group may have lower CD4^+^ T cell counts and poor ART effects on reducing anti-CD4 antibody levels due to late treatment. Thus, it is more difficult to gain significant effect of ART on anti-CD4 IgGs and CD4+ T-cell counts. In addition, the design and data analyses may affect the results. We will conduct prospective cohorts or a more complicated experimental design in future research, such as larger sample size and longer ART. Furthermore, the detailed mechanisms and pathologic role of anti-CD4 IgG after early treatment needs to be further investigated, including how anti-CD4 IgG mediate CD4^+^ T-cell death via ADCC after early ART in AHI patients.

In summary, we first reported the dynamic change of anti-CD4 IgG in AHI individuals following early or delayed ART and showed that early ART but not delayed ART normalized anti-CD4 IgG levels. Plasma anti-CD4 IgG levels after 48W of ART were inversely correlated with post-ART CD4^+^ T-cell counts in early treated AHI individuals, suggesting that high levels of anti-CD4 IgG during immune recovery may contribute to blunt CD4^+^ T-cell recovery after early ART.

## Data Availability Statement

All datasets generated for this study are included in the article/supplementary material.

## Ethics Statement

This study has been approved by the Beijing Youan Hospital Research Ethics Committee and all participants provided written informed consent. The methods were carried out in accordance with the approved guidelines.

## Author Contributions

ZL, BS, WJ, and HW conceived and designed the experiments. AS, LL, WX, and TZ collected the sample information, contributed to reagents and materials. AS, ZL, ZwL, XL, RW and ZW performed the experiments and analyzed the data. AS, ZL, BS, WJ, and HW wrote the manuscript. All authors read and approved the final manuscript.

## Funding

This work was supported by the National Natural Science Foundation of China (NSFC)-NIH Biomedical collaborative research program (81761128001 to HW), the National Institutes of Allergy and Infectious Diseases (AI1288864 to WJ), the NSFC (81772165 and 81974303 to BS; 81571973 to HW; 81501731 to ZL; 81501732 to XL), the National 13^th^ Five-Year Grand Program on Key Infectious Disease Control (2017ZX10202102-005-003 to BS; 2017ZX10202101-004-001 to TZ; 2018ZX10301-102-002 to ZL), the Beijing Municipal of Science and Technology Major Project (D161100000416003 to HW), and the Beijing Key Laboratory for HIV/AIDS Research (BZ0089). The funders had no role in study design, data collection and analysis, decision to publish, or preparation of the manuscript.

## Conflict of Interest

The authors declare that the research was conducted in the absence of any commercial or financial relationships that could be construed as a potential conflict of interest.
